# Screening for extremely rare pathogenic variants of monogenic diabetes using targeted panel sequencing

**DOI:** 10.1007/s12020-021-02753-7

**Published:** 2021-05-21

**Authors:** Tomasz Płoszaj, Karolina Antosik, Paulina Jakiel, Agnieszka Zmysłowska, Maciej Borowiec

**Affiliations:** Department of Clinical Genetics, Medical University of Lodz, Pomorska 251, 92-213 Lodz, Poland

**Keywords:** Next-generation sequencing, Monogenic diabetes, Rare variant, MODY

## Abstract

**Aims:**

Maturity‐onset diabetes of the young (MODY) is one of the rare monogenic forms of diabetes. To date, about 12 genes in the scientific literature are closely related to the occurrence of the disease phenotype. However, there is still a high prevalence of undiagnosed cases of so-called MODY-X whose genetic background is still unknown.

**Methods:**

We performed tNGS for 523 patients with suspected MODY. Next 357 selected patients, in whom no damaging variants were found in 12 major genes causing MODY, were screened for the presence of pathogenic variants in four candidate genes (*MNX1, RFX6, NKX2.2*, and *NKX6.1*). All data were generated in one tNGS sequencing reaction and confirmed by Sanger sequencing.

**Results:**

In total, we selected five potentially damaging variants, in eight patients, in *RFX6, NKX2.2*, and *NKX6.1* genes. Four of them have never been described in literature before. The frequency of occurrence of two of them in the *RFX6* gene significantly differed in relation to the healthy population. The analysis of segregation in the family did not reveal that they were the only cause of the disease phenotype.

**Conclusions:**

The very-rare variants indicated in this study show that this type of research on large population groups may help in the future for better understanding and more accurate diagnostics of extremely rare forms of MODY.

## Introduction

Maturity‐onset diabetes of the young (MODY) is a group of rare monogenic diabetes (1–2%), genetically determined, resulting in changes in the functioning of insulin secretion of pancreatic beta cells [1]. The phenotype is characterized by early onset of hyperglycemia and negative autoantibodies characteristic for type 1 diabetes mellitus (T1DM) [2]. To date, about 13 genes have been indicated that are most often associated with the presence of MODY phenotype [3]. Due to the huge genetic diversity underlying this disease and the methodological difficulties associated with determining changes in some genes, the latest sequencing techniques seem promising for rapid diagnosis and the search for yet unknown variants in other MODY candidate genes. There are several screening reports showing that a large proportion of patients (~60%) have so-called MODY-X due to other changes than those of the most-analyzed genes like *GCK* or *HNF1A* [4, 5]. Some scientific reports suggest that, among others, four genes such as *MNX1, RFX6, NKX2.2*, and *NKX6.1* can be responsible for the development of some rare cases of MODY diabetes [1, 6–9]. These genes were included in a targeted panel of genes routinely sequenced in our laboratory from suspected MODY patients. The aim of this study was to identify potentially new damaging variants in the above-mentioned four gene candidates by analyzing large cohort of the MODY patients, which lacks detrimental mutations in any of the 12 main MODY genes (*HNF4A, GCK, HNF1A, PDX1, HNF1B, NEUROD1, CEL, INS, ABCC8, KCNJ11, APPL1*, and BLK).

## Material and methods

### Subjects

Hyperglycemia or diabetes mellitus of unspecified etiology was diagnosed in all 523 patients according to the World Health Organization definition [10]. Body mass index (BMI) was defined in adult patients as body weight divided by the square of body height (kg/m^2^). In children, the BMI value was additionally related to the percentiles adjusted for age and gender. In all patients, the following parameters were analyzed: HbA1c value (%) as an indicator of chronic hyperglycemia, fasting C-peptide value (ng/mL) as a marker of preserved insulin secretion, in pediatric patients at least two T1DM-specific antibodies and in adults at least glutamic acid decarboxylase antibodies to differentiate from latent autoimmune diabetes in adults.

**Fig. 1:**
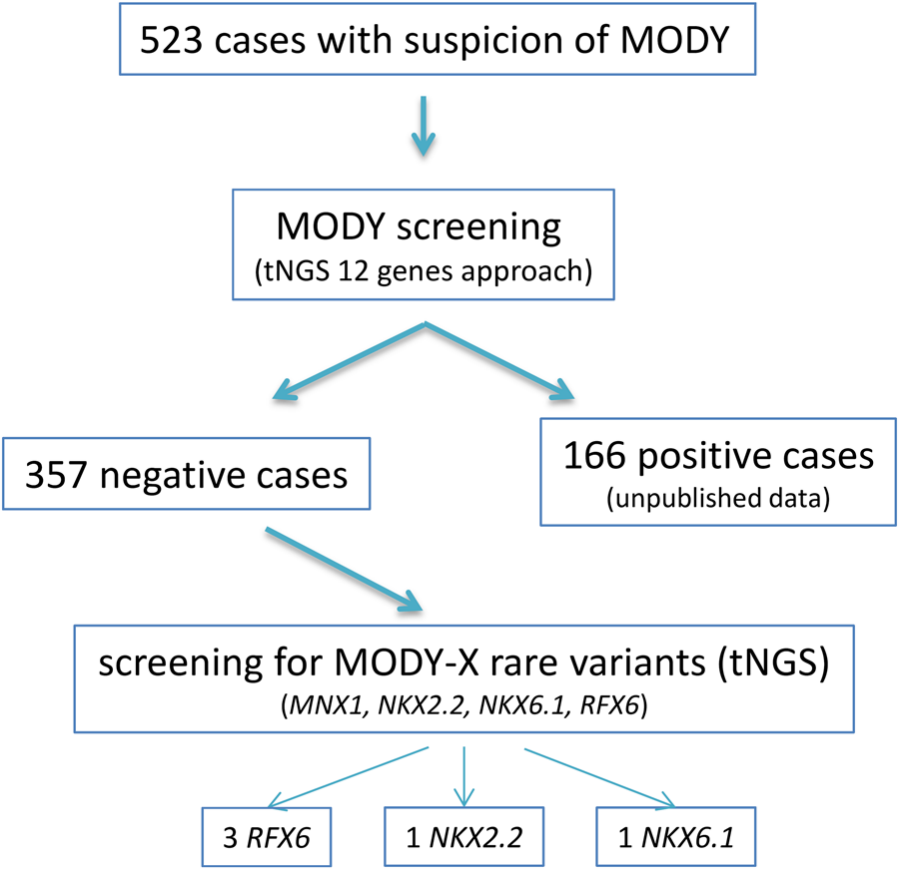
Flowchart of the study cohort selection

Initially, exon variants were analyzed in 523 patients in 12 main genes that cause MODY (*HNF4A, GCK, HNF1A, PDX1, HNF1B, NEUROD1, CEL, INS, ABCC8, KCNJ11, APPL1,* and *BLK*) by the tNGS analysis (unpublished data). Then, a cohort of 357 patients who were negative for the presence of pathogenic variants in the 12 studied genes was analyzed again in search for the possible and detrimental mutations in four candidate MODY genes (*MNX1, RFX6, NKX2.2,* and *NKX6.1*). Data for both analyses were derived from the same samples that were processed according to the same methodology involving tNGS library preparation and sequencing. Flowchart of the study cohort selection is presented in Fig. 1.

### Library preparation and sequencing reaction

DNA isolation was performed from peripheral blood with a semi-automatic device from the Promega Maxwell^®^ RSC Instrument. The library was prepared in accordance with the Agilent SureSelectQXT Target Enrichment protocol using a custom gene panel in accordance with the manufacturer’s instructions. Paired‐end sequencing was performed in a NextSeq550 System, Illumina (2 × 150 bp).

### Bioinformatic analysis

Raw FASTQ files were (GRCh37/hg19) mapped using the BWA-MEM alignment algorithm [11] and duplicate reads were removed using Picard tool [12]. Analysis of target regions, mapping quality was performed using BEDTools (coverage statistics) [13]. FreeBayes algorithm [14] was used for the variant calling and functional annotation process performed using ANNOVAR [15]. All above bioinformatic analysis was performed on the local instance of galaxy [16] or on-line web-based platforms. Statistical analyses were performed using MedCalc for Windows, (MedCalc Software, Ostend, Belgium). The analysis of conservativeness in the chosen variants was performed in MEGA X: molecular evolutionary genetics analysis [17]. The considered sequences were derived from three species (*Gorilla Gorilla*, *Mus musculus*, and *Canis lupus familiaris*).

### Variant selection criteria

Assigned variants of the studied genes were filtered with respect to frequency of the base gnomAD [18], European population below 0.01. Subsequently, if the filtered variants have determined their harmful potential from a database SIFT [19] and PROVEAN [20], only those variants were selected that in at least one database was marked as damaging. In our study, only variants with the following consequences: loss of function/null variants (indels, stop-gain, splice site) or nonsynonymous were considered. The sequencing quality of the indicated variants had to meet the minimum coverage requirement of 20× coverage and allelic balance ~50%. The selected variants in the discovery cohort were also compared with a group of patients (*n* = 166), who had confirmed diseases genetically determined by other genome changes, in order to verify that the results were not caused by an internal method error or data processing.

### Variant confirmation

Selected variants were amplified using five primer pairs. The sequences of all primer pairs used in the analysis are shown in Table S2. PCR was performed in 25 μl with 1 μl of patient DNA, using standard reagents including HotStarTaq DNA Polymerase (Qiagen), with a profile of 35 cycles and annealing at 59 °C. Approximately 3 μl of the amplicons were visualized on 2.8% agarose gel. After enzymatic purification (EPPiC, A&A Biotechnology), PCR products were extended using the BigDye^®^ 3.1 termination-ready reaction mix. Each sequencing reaction (20 μl) contained 4 μl of BigDye^®^ mix, 30 ng of a primer and 50–70 ng of the amplicon. Extension products were purified (BigDye XTerminator, Thermo Fisher Scientific) and analyzed using an ABI Prism 3130™ Genetic Analyzer. Sequences were edited and analyzed using BioEdit and MEGA 4: molecular evolutionary genetics analysis [17].

## Results

We identified five potentially pathogenic variants in a heterozygous form in the negative patient cohort. Three of them were in the *RFX* gene and one in the *NKX6.1* and *NKX2.2* genes. Details of the selected variants are contained in Table S1. In the *MNX1* gene, no variant was identified that could potentially cause MODY in the studied population.

The selected variants were identified in eight patients, including three men (37.5%), who were heterozygous in terms of age at the time of genetic examination (from 8.1 to 57.3 years with the median age of 17.7 years. Detailed clinical characteristics of the patients and their parents are summarized in the Supplementary Information and in Table S1. All patients had hyperglycemia or diabetes recognized on average at 17.3 years of age. All patients had a normal BMI index, no overweight or obesity was observed.

For two variants of the *RFX6* gene for which the comparative data were available, the calculated odds ratio showed statistically significant differences compared to the healthy population (Table 1). In the pool of 166 patients who were diagnosed with MODY due to changes in other genes, none of the described changes was noted, which indicates that the selected changes are not the result of a methodological error or artifact of bioinformatic processing. In addition, the presence of all five variants was confirmed by direct Sanger sequencing in patients. The conservativeness analysis of the chosen variants indicate that they all are conserved among various species, except one variant in *RFX6* gene. More detailed information can be found in Table S1.

**Table 1:** Frequency of chosen heterozygous variants in the study cohort and control populations

Gene	Patient ID	AA change	Discovery cohort (%)	gnomAD EUR (%)	Odds ratio (95% CI)	*P* value	ACMG classification
*RFX6*	1,2,3	Thr169Thr (splice region)	3 (0.84)	41/129048 (0.032)	26.67 (8.2191–86.5112)	<0.0001	Likely pathogenic
*RFX6*	4,5	Ser854Leu	2 (0.56)	104/129136 (0.081)	6.99 (1.7184–28.4321)	0.0066	Uncertain significance
*RFX6*	6	Gly194Glu	1 (0.28)	–	–	–	Likely pathogenic
*NKX2-2*	7	Ala138Glu	1 (0.28)	–	–	–	Uncertain significance
*NKX6-1*	8	Ser180Arg	1 (0.28)	–	–	–	Uncertain significance

## Discussion

We have analyzed the presence of potentially pathogenic variants in four candidate genes in 357 unrelated patients from Poland who have been suspected of MODY-X diabetes. All five selected variants occurred in a heterozygous form, which is not a typical inheritance form for MODY. However, for all considered genes, it has been reported in the literature that there are cases where a recessive form of inheritance of variants of these genes has been observed [6, 9]. Due to the presence of rare genetic variants identified in patients of different age, we combined them into the one group. It is worth noting that the cutoff points for hyperglycemia and diabetes are the same for children and adults, as same as the HbA1c value, antibody titer, and insulin secretion value. BMI was calculated differently for adults and children and was taken into account in the characteristics of the study group.

Three of the selected variants are present in the *RFX6* gene, which affects insulin secretion in beta cells of the pancreas by modulating intracellular calcium concentration homeostasis [21]. Several variants have already been described in the literature that are associated with the MODY diabetes phenotype [6, 22]. Two changes indicated in this study are newly described variants (p. Gly194Glu, p. Thr169Thr), while one (p. Ser854Leu) has already been reported in ClinVar databases as probably related to carbohydrate metabolism disorders. The highest odds ratio (26.67) shows a synonymous change that is located in the splice region of the fourth exon, which may affect the folding process of protein. Such a high OD value clearly indicates the increased incidence of this variant in the discovery cohort, which suggests that it may be causative in MODY diabetes. Interestingly, this particular nucleotide is conserved among various species although it does not affect the amino acid sequence in the protein. However, the conducted segregation analysis for two families did not confirm that it was directly associated with the disease phenotype (Fig. S1).

The second variant, which frequency significantly differs from the control group (OD: 6.99), is the nonsynonymous variant in the fourth exon (p. Ser854Leu). In this case, the difference in frequency is no longer as drastic and in addition, as the only change identified, it also has a significantly higher frequency in the cohort of people with type 2 diabetes mellitus [23]. In the ClinVar database, it has the status of “conflicting interpretations of pathogenicity” and is associated with the phenotype of “monogenic diabetes” and “Mitchell–Riley syndrome”. This indirectly confirms that in fact this variant somehow affects the disorder of diabetes metabolism in patients. However, the segregation analysis for two families did not confirm that it was directly associated with the phenotype disease (Fig. S1). Moreover, the analysis of conservativeness did not indicate consistency of this variant among various species, therefore its pathogenicity cannot be confirmed.

The last variant of the *RFX6* gene located in the fifth exon (p. Gly194Glu) is a change never described before for which there is no data on the frequency of occurrence in the gnomAD/ExAC databases [18]. Both predictive programs used give very-high values (>0.9) indicating the damaging nature of this change. In this case, due to random reasons, unfortunately, it was not possible to perform a family segregation analysis and inference is based solely on in silico prediction.

Two new, never before described, potentially pathogenic variants have also been identified in the *NKX2-2* (p. Ala138Glu) and *NKX6-1* (p. Ser180Arg) genes. According to the literature, these genes can also affect the functioning of pancreatic beta cells [7, 24] and have been phenotypically identified as causing diabetes [8, 9]. The analysis of segregation in the family was only possible for variant in the *NKX6-1* gene and did not confirm that this change was directly causative and correlated with diabetes metabolism disorder in this case (Fig. S1).

In the segregation analyses carried out in three of the five variants described, we were unable to observe a direct genotype–phenotype link. Unfortunately, due to random reasons and those resulting from the fact that the indicated variants are extremely rare in population, we were not able to verify them on a larger group of patients, which can be treated as a limitation of the study. The lack of extensive analysis of phenotypes among the other family members limits the ability to precisely indicate the influence of the selected gene variants on the MODY phenotype. It is possible that, similarly to the earlier reports about for example heterozygous variants in the *RFX6* [6] gene, the penetration of the described changes is not complete and therefore we do not see a direct correlation of the genotype–phenotype in these specific cases. On the other hand, their uniqueness, and in two cases, a statistically significant increase in the prevalence in our discovery cohort seems to clearly suggest this. In the case of changes for which there is no comparative data available on the prevalence, their damaging nature was indicated by the predictive programs used. Moreover, the high conservativeness in the studied variants (except Ser854Leu in *RFX6* gene) was confirmed among three species, also in synonymous splice site in *RFX6* gene. However, the final confirmation of their effect on the MODY disease phenotype requires testing on a larger group of carriers. The very-rare variants indicated in this study show that this type of research on large population groups may help in the future in better understanding and more accurate diagnostics of extremely rare forms of MODY.

Furthermore still a very large proportion of patients remains in our discovery cohort in whom the cause of diabetes has not been determined. Concluding this shows that the tNGS approach and searching for changes only in specific genes is an ineffective way of MODY diagnosis in some cases. For these patients and their families, the whole-exome sequencing or whole-genome sequencing should be performed and maybe such a comparison would allow more accurate diagnostics of these difficult MODY genotypes.

## Supplementary information

Supplementary Information
