# Supplementary material for: Screening for extremely rare pathogenic variants of monogenic diabetes using targeted panel sequencing
Source: Endocrine. 2021 May 21;73(3):752–7. doi: 10.1007/s12020-021-02753-7 (PMC8325655; doi:10.1007/s12020-021-02753-7)
Supplement: Supplementary file 1 — Supplementary Information [file 12020_2021_2753_MOESM1_ESM.docx]

**Clinical characteristics of the selected patients**

In all patients, no insulin secretion disorders were found, with the exception of patient #7, in whom at the time of diabetes diagnosis a decreased C-peptide level of 0.37 ng/ml was observed (normal range: 0.9-4.0 ng/ml), and therefore insulin therapy was implemented. Moreover, in patient #7, two positive antibodies characteristic of T1DM were observed, which additionally indicated the necessity of using insulin therapy. Currently, after several years of diabetes duration, unlike in the T1DM, C-peptide concentration has normalized in this patient and the daily subcutaneous insulin requirement is low at 0.14 IU/kg body weight. In another patient #4, three antibodies characteristic of T1DM were also observed, but in the absence of insulin secretion disorders and only fasting hyperglycemia, insulin therapy was not decided. Interestingly, this patient also has additional disorders such as IgA deficiency, history of vesicoureteral reflux, kidney cyst, sinus tachycardia and mitral valve prolapse. The remaining patients were negative for T1DM-specific autoantibodies and are treated only with diet and/or oral hypoglycemic drugs such as sulphonylurea derivatives (SUR) or metformin. In the case of patient #8, in which, apart from diabetes, a heart defect in the form of a bicuspid aortic valve was found, a combination of insulin therapy (0.2 IU/kg) and metformin was implemented.

**Table 1**. Clinical characteristics of the study group.

| **Patient**  **ID** | **Gender (F/M)** | **Age at study time (years)** | **IFG**  **/Diabetes** | **Age at hyperglycemia/diabetes onset (years)** | **HbA1c at study time (%)** | **Other symptoms** | **Treatment** | **Hyperglycemia (H)/diabetes (DM) in parents of the patient** |
| --- | --- | --- | --- | --- | --- | --- | --- | --- |
| #1 | M | 25.6 | Diabetes | 22.7 | 6.4 | No | Metformin | Mother-DM |
| #2 | M | 16.9 | IFG | 14.2 | 5.1 | No | Diet | No |
| #3 | F | 17.9 | IFG | 13.1 | 5.6 | No | Diet | Mother-H |
| #4 | F | 19.2 | IFG | 15.2 | 5.8 | IgA deficiency,  vesicoureteral reflux, kidney cyst, tachycardia, heart defect | Diet | No |
| #5 | F | 8.1 | IFG | 2.1 | 5.1 | No | Diet | No |
| #6 | M | 57.3 | Diabetes | 47.2 | 6.4 | No | SUR | Mother and father- DM |
| #7 | F | 16.5 | Diabetes | 11.1 | 6.0 | Hashimoto disease, scoliosis | Insulin | Mother-GDM |
| #8 | F | 17.5 | Diabetes | 13.2 | 6.8 | Heart defect | Metformin, insulin | Father-DM |

**IFG** – impaired fasting glycemia; **SUR** – sulphonylurea; **GDM**-gestational diabetes mellitus

**Figure 1:** Pedigrees of families with heterozygous variants who were identified in discovery cohort. Genotype is shown underneath each symbol; M and N indicate mutant and wild-type alleles, respectively. Below is the genotype, age of diabetes onset in years, duration in years. Squares represent male family members, and circles represent female sex. Black-filled symbols denote patients with diabetes, an arrow denotes the proband in the family.


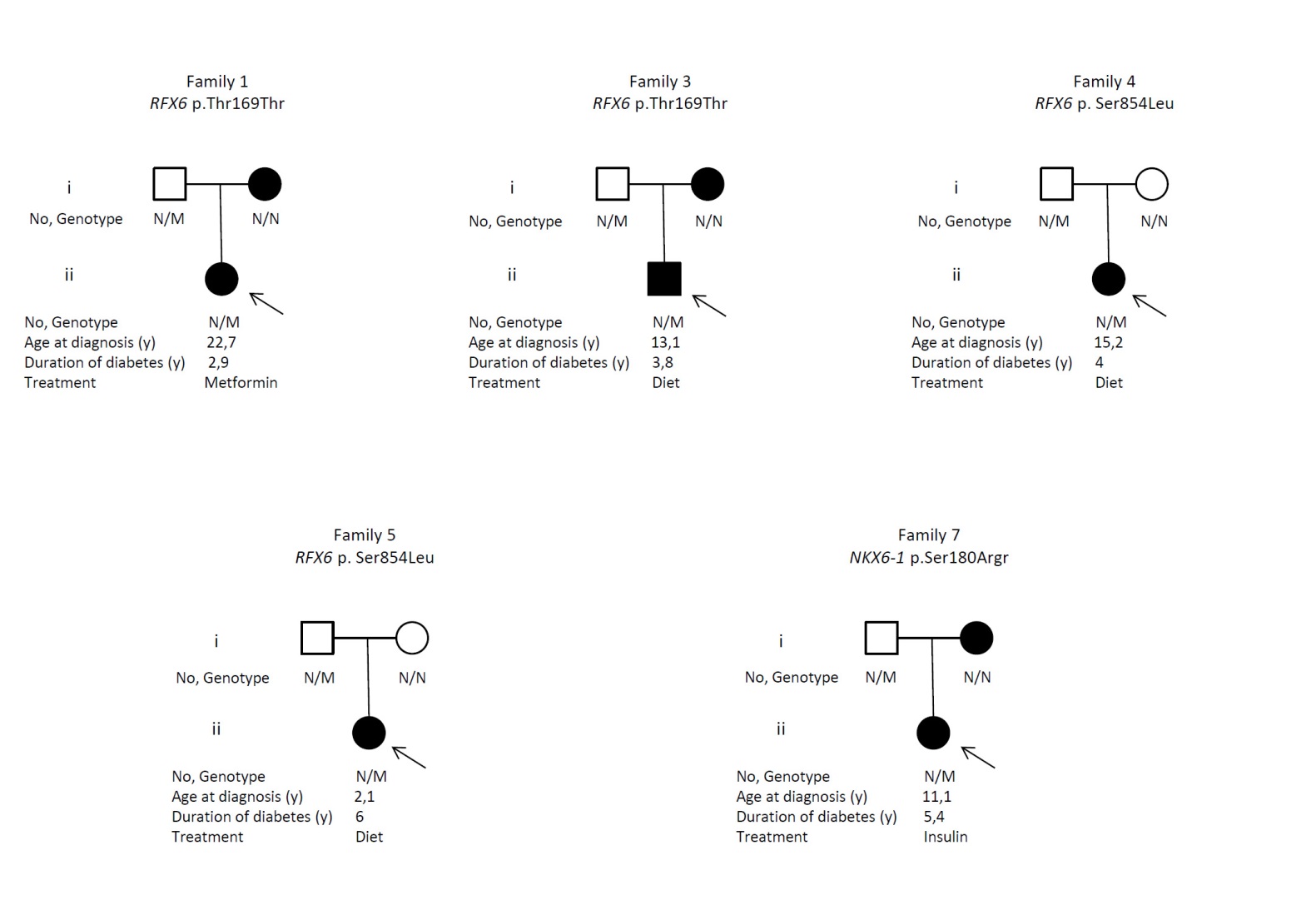


| **Chromosome coordinates** | **Gene** | **Exonic function** | **SNV/AA change** | **dbSNP** | **ClinVar_DIS** | **SIFT converted rankscore** | **SIFT prediction** | **PROVEAN converted rankscore** | **PROVEAN prediction** | **Conservativeness analysis** |
| --- | --- | --- | --- | --- | --- | --- | --- | --- | --- | --- |
| chr6:117215164 | RFX6 | nonsynonymous SNV | NM_173560:EX:5:c.581G>A:p.Gly194Glu | . | . | 0.912 | D | 0.928 | D | *G.gorilla*:G; *M.musculus*:G;  *C. lupus*:G **(CONSERVED)** |
| chr6:117203532 | RFX6 | synonymous SNV (splice region) | NM_173560:EX:4:c.507A>T:p.Thr169Thr | rs151067974 | . | . | . | . | . | *G.gorilla*:A; *M.musculus*:A;  *C. lupus*:A **(CONSERVED)** |
| chr6:117250084 | RFX6 | nonsynonymous SNV | NM_173560:EX:18:c.2561C>T:p.Ser854Leu | rs201522681 | Monogenic diabetes, Mitchell-Riley syndrome | 0.461 | D | 0.314 | N | *G.gorilla*:C; *M.musculus*G;  *C. lupus*: no such exon **(NOT CONSERVED)** |
| chr20:21492970 | NKX2.2 | nonsynonymous SNV | NM_002509:EX:2:c.413C>A:p.Ala138Glu | . | . | 0.721 | D | 0.779 | D | *G.gorilla*:A; *M.musculus*:A;  *C. lupus*:A **(CONSERVED)** |
| chr4:85418842 | NKX6.1 | nonsynonymous SNV | NM_006168:EX:1:c.540C>G:p.Ser180Arg | . | . | 0.564 | D | 0.270 | N | *G.gorilla*:C; *M.musculus*:C;  *C. lupus*:C **(CONSERVED)** |

**Table 2.** Genetic characteristics of MODY candidate variants.

**Table 3.** Primer sequences used for variant verification

| **Gene target** | **Primer sequence 5'-3'** | **Chromoseome coordinates** | **Product length** |
| --- | --- | --- | --- |
| RFX6ex4_F | TGCCTCTGCTGATGTATTGC | chr6:117203353+117203707 | 355bp |
| RFX6ex4_R | GCTTCATCAAGAGAATACCTGGTT |  |  |
| RFX6ex18_F | GCACAAATCCAGTTCTGTAGCA | chr6:117249769+117250130 | 362bp |
| RFX6ex18_R | GACTGGAGTTCGACATGCAA |  |  |
| NKX6ex1_F | AAGCGAGAATCCCTTTCTGG | chr4:85418533+85419226 | 694bp |
| NKX6ex1_R | CTCGTCGTCGTCCTCCTC |  |  |
| NKX-2.2ex2_F | GGTGTGCTGTCGGGTACTG | chr20:21492593+21493097 | 505bp |
| NKX-2.2ex2_R | CCTCAGGACTCAAGCTCCAA |  |  |
| RFX6ex5_F | CATGTTGAATCACCCAATTTGT | chr6:117214795+117215371 | 577bp |
| RFX6ex5_R | TCATCCATACAAAGCGGACA |  |  |
